# *In Vivo* Study of Dynamics and Stability of Dendritic Spines on Olfactory Bulb Interneurons in *Xenopus laevis* Tadpoles

**DOI:** 10.1371/journal.pone.0140752

**Published:** 2015-10-20

**Authors:** Yu-Bin Huang, Chun-Rui Hu, Li Zhang, Wu Yin, Bing Hu

**Affiliations:** Chinese Academy of Sciences Key Laboratory of Brain Function and Disease, and School of Life Sciences, University of Science and Technology of China, Hefei, Anhui Province, P. R. China; University of South Alabama, UNITED STATES

## Abstract

Dendritic spines undergo continuous remodeling during development of the nervous system. Their stability is essential for maintaining a functional neuronal circuit. Spine dynamics and stability of cortical excitatory pyramidal neurons have been explored extensively in mammalian animal models. However, little is known about spiny interneurons in non-mammalian vertebrate models. In the present study, neuronal morphology was visualized by single-cell electroporation. Spiny neurons were surveyed in the *Xenopus* tadpole brain and observed to be widely distributed in the olfactory bulb and telencephalon. DsRed- or PSD95-GFP-expressing spiny interneurons in the olfactory bulb were selected for *in vivo* time-lapse imaging. Dendritic protrusions were classified as filopodia, thin, stubby, or mushroom spines based on morphology. Dendritic spines on the interneurons were highly dynamic, especially the filopodia and thin spines. The stubby and mushroom spines were relatively more stable, although their stability significantly decreased with longer observation intervals. The 4 spine types exhibited diverse preferences during morphological transitions from one spine type to others. Sensory deprivation induced by severing the olfactory nerve to block the input of mitral/tufted cells had no significant effects on interneuron spine stability. Hence, a new model was established in *Xenopus laevis* tadpoles to explore dendritic spine dynamics *in vivo*.

## Introduction

The dendritic spine, a small membranous protrusion from a neuron’s dendrite, is a postsynaptic structure that stores synaptic strength and transmits electrical signals within neural circuits. Dendritic spines are dynamic, and their turnover and stability are vital for development, memory, and learning [1–14]. To decipher complex brain functions and disorders, non-mammalian vertebrates such as zebrafish and *Xenopus* offer unique advantages and simplified but significant structural and functional homology to the human central nervous system and have been used to study synaptogenesis and dendrite development [15–25]. However, few studies on dendritic spine plasticity, spinogenesis, or spine pruning have been conducted in such model systems. Most knowledge about spine morphology and dynamic features has been gained from studies on mammalian brains in barrel, visual, and motor cortices [4, 5, 10, 26].

With the exception of some spine-like structures, dendritic spines are not found in zebrafish [16, 27]. The *Xenopus* visual system has been widely used for *in vivo* studies [28–33]; however, tectal neurons are not spiny neurons. Granule cells in the olfactory bulb (OB) of *Xenopus* are the only reported spiny neurons according to morphology in slices [31, 34]. Is this the only spiny neuron with typical morphology in *Xenopus*? What are the dynamic features of this model? In this study, we examined the distribution of spiny neurons in the *Xenopus* brain, as well as dynamic changes and stability of spiny neurons in the OB during development and after odor deprivation.

## Materials and Methods

### Animals

Adult *X*. *laevis* females (Nasco, Fort Atkinson, WI) were primed with human chorionic gonadotropin (Sigma-Aldrich, St Louis, MO) to induce egg laying. Oocytes were fertilized *in vitro*. Tadpoles were maintained in a modified rearing solution (60 mM NaCl, 0.67 mM KCl, 0.34 mM Ca(NO_3_)_2_, 0.83 mM MgSO_4_, 10 mM HEPES adjusted to pH 7.4, and 40 mg/L gentamycin) with 0.001% phenylthiocarbamide (PTU) added to prevent melanocyte pigmentation under filtered illumination in 12 h dark/light cycles.

During labeling and imaging, tadpoles were anesthetized with 0.02% MS-222 (Tricaine methanesulfonate, Finquel, Argent Laboratories, Reymond, WA). Staging was performed according to Nieuwkoop and Faber [35]. All animal manipulations were conducted in strict accordance with the guidelines and regulations set forth by the University of Science and Technology of China (USTC). Furthermore, the protocol was approved by the Committee on the Ethics of Animal Experiments of the USTC (Permit No.: USTCACUC1102012).

### Electroporation for single-neuron labeling

Morphologies of spiny and non-spiny neurons were distinguished by single-cell electroporation of dye as previously described [36, 37]. Borosilicate pipettes (tip diameter 1 μm, 6–10 MΩ resistance) were filled with plasmid (0.8–1.2 μg/μL) and Alexa Fluor 594 (Molecular Probes, USA) in extracellular solution. For dye labeling, pipettes were filled with Fluor 568-conjugated dextran (MW 10,000, Molecular Probes, USA) dissolved in extracellular solution. Patch pipettes were mounted to an electrode holder attached to a micromanipulator (Siskiyou, USA). The electrode was inserted through the skin of the tadpole, which was embedded in low melting point agarose, and the electrode tip was placed near the soma of interest. Dye was electroporated into neurons using 50 negative (positive for dextran-conjugated dye) square 10–12 V pulses of 0.5 ms duration at a frequency of 100 Hz generated by an Axoporator 800A (Axon Instruments, USA).

Neurons located in the OB, telencephalon, and tectum were randomly labeled. For clear imaging, the depth of the labeled neurons was 0–100 μm under the skin. Only well-labeled neurons with clear processes were selected as samples for imaging. To confirm the positions of labeled neurons in the brain and detailed dendrite morphology, images were scanned with 10× and 60× objectives, respectively.

### *In vivo* expression of PSD95-GFP and dendritic spine labeling

To visualize simultaneously the postsynaptic specializations and olfactory neuron morphology, OB neurons were co-transfected with the expression plasmids PSD95-GFP and DsRed2. PSD95-GFP was constructed and provided by Dr. D. Bredt (University of California, San Francisco, CA) [38]. The full sequence of PSD95 was generated via PCR and subcloned into the HindIII and EcoRI sites of the GW1 expression vector with a cytomegalo virus (CMV) promoter (British Biotechnology, UK). GFP was subcloned in-frame at the C terminus of PSD95 at the EcoRI site [38]. DsRed2 is a plasmid with a CMV promoter that drives the expression of a variant of *Discosoma sp*. red fluorescent protein (Clontech, Cat. No. 632430, USA).

Plasmids of PSD95-GFP and DsRed2 (0.8–1.2 μg/μL) were mixed for co-electroporation when the tadpoles reached stage 45/46. After 48 h, tadpoles with distinct neurons expressing DsRed2 throughout their dendritic arbor and those with punctuate PSD95-GFP labeling were selected for imaging. Approximately 80–90% of the transfected neurons expressed both plasmids [19]. Given that expression of PSD95-GFP might promote synaptic maturation [39], only DsRed2- or GFP-transfected neurons were used for analyses of spine dynamics and stability. The PSD95-GFP and DsRed2 double-labeled neurons were selected only for colocalization analysis of PSD95-GFP and classification of the different types of spine morphology.

### Neural input deprivation

*X*. *laevis* tadpoles (stage 46/47) were anesthetized with 0.02% MS-222. A small fissure on the skin over the OB was made using iridectomy scissors under a binocular stereo microscope. Using a fine #5 forceps (FST, Switzerland), the olfactory nerve was completely severed to block sensory input to the OB. The surgically treated individuals were reared in a modified rearing solution for recovery. To confirm that the olfactory nerves were severed, a small amount of 0.1% DiI (N22880, Invitrogen, USA) was pressure-injected into the OB using a microinjector (PicoSpitzer III, USA). After 24 h, both fully retrograde-labeled olfactory nerves were severed and immediately imaged using a fluorescence stereo microscope (Olympus SZX16, Japan).

### *In vivo* time-lapse imaging

Tadpoles with single labeled spiny neurons located in the posterior part of the OB were selected for *in vivo* imaging. The imaging procedure was similar to previously used protocols [30, 40–43]. An imaging chamber was constructed of 2% agar with wells in a petri dish. The anesthetized tadpole was placed in a well and further immobilized by low melting point agarose (1% in rearing solution). The petri dish was then filled with dilute anesthetic solution perfused with air. After imaging, the tadpole was removed from the chamber and returned to normal rearing solution to recover. Viability of the animals was ensured through heart rate and recovery time. Imaging was performed under a 60× water immersion objective lens on Olympus FV1000 confocal microscope (Olympus, Japan) equipped with Argon and He-Ne lasers. Thin (1.0 μm) overlapping optical sections encompassing the entire dendritic arbor were collected below saturation levels, with minimal gain and contrast enhancements. For time-lapse imaging, stacks were collected every 15 min over a 60-min period to evaluate short-term dynamics, and every 2 h over a 6-h period and at 24 h for dynamic analysis. After surgery to block neural input, confocal images were obtained at 0 h, 2 h, and 24 h.

### Image processing and analysis

Well-isolated OB spiny neurons with 6 to 9 dendrites were selected, and the entire length (rather than a portion) of the dendrite was used for quantitative analysis. For spine categorization, classification was based on approximate measurements made on the magnified projected images according to previously described criteria [12, 44]. Filopodia are long, thin protrusions that have a hair-like morphology. Thin spines have small heads and narrow necks, and the diameter of the head and neck are of similar magnitudes: their length is greater than neck diameter. Stubby spines have no obvious constrictions between the head and the dendritic shaft. The diameter of its neck is nearly equal to the total length. Mushroom spines have large heads and narrow necks. Branched spines have more than one head. Filopodia and thin spines are classified as small spines, whereas stubby and mushroom spines are considered large spines [13].

Three-dimensional (3D) reconstruction of the confocal image stacks was performed and a median filter (3 × 3 × 1) was applied using Imaris 7.0 (Bitplane, Switzerland). The 3D object could be magnified and rotated in all directions. Traces of spines and synapses were detected using 3D images. Under these conditions, we were able to obtain larval spine data on the Z-axis or behind the overlap region by carefully rotating the images.

The Imaris Filament module was used to define and classify the 4 different types of dendritic spines. All dendritic arbors of a single neuron were traced using the AutoPath mode of the Filament module. The dendritic spines were traced based on the dendritic framework using the Manual option. Each spine type was assigned a unique color. Branched spines were not included in the final statistical data due to the limited number of cases.

The digital 3D reconstruction images contained two color channels. DsRed2 labeled the olfactory spiny neurons and dendritic arbors, whereas PSD95-GFP labeled the postsynaptic spine specializations. The overlaps of these two channels were analyzed to determine the identity and position of PSD95-GFP along the dendritic arbor. PSD95-GFP-labeled puncta with areas between 0.5 and 1.0 μm^2^ and green channel pixel intensity values between 50 and 255 were considered single synapses. Discrete PSD95-GFP-labeled puncta exhibited median pixel values 2 to 3 times greater than the median pixel values of the background non-puncta GFP within the same dendritic arbor. In the data analysis, similar ratios were maintained for every dendritic arbor analyzed throughout the observation period. Spines were automatically detected using the Spots module, and spots that were not exactly located inside the spines were manually deleted.

### Data analysis

A total of 21 tadpoles each with single-labeled neuron were selected for imaging and spine analysis. To minimize injury, the number of electroporated neurons was limited in each OB, which is less efficient; the sample acquisition was difficult because of the selection criteria and labeling efficiency. Four neurons with 9 individual dendrites were used for the co-localization analysis of PSD95-GFP. Four neurons with 9 dendrites were used for the 15-min interval short-term observation, and 4 neurons with 7 dendrites were used for the 2-h interval and 24-h observations. Ten neurons with 16 dendrites were used for the sensory deprivation analysis. The neurons analyzed in the transformation data were those from the 15-min and 2-h observations. In the data analysis, all the dendrites from a neuron are pooled as an independent sample for statistics. Several parameters were measured to obtain detailed information on the dynamics of the 4 types of dendritic spines at each observation interval: the number of spines added and eliminated, and the number of spines maintained from one observation to the next. The maintained spines were classified as stable, whereas the added and eliminated spines were considered dynamically changed. The percentages of the stable, added, and eliminated spines were calculated. Spine stability was defined as the percentage of stable spines compared with previously observed ones. Spine addition was defined as the percentage of added spines compared with the next observed ones. Spine elimination was defined as the percentage of eliminated spines compared with previously observed ones. From the starting imaging to the next, an eliminated spine may disappear directly, or transform into other spine types. Therefore, the percentages of spines that appeared or disappeared, as well as those of each type that transformed into the other 3 types, were calculated. The percentages of PSD95-GFP puncta present in the 4 types of spines were also calculated. Data are presented as percent increases from the initial observation interval to each subsequent interval or as percent increases for each 2-h observation interval.

Two-sample unpaired *t*-tests and one-way ANOVA with Tukey’s multiple comparison tests (GraphPad Prizm 4.0) were used for statistical analysis of the data at *P < 0.05, **P < 0.01, and ***P < 0.001 significance levels. Data are presented as mean ± standard error of the mean (SEM).

## Results

### Distribution of spiny neurons in the *Xenopus* brain

**Fig 1:**
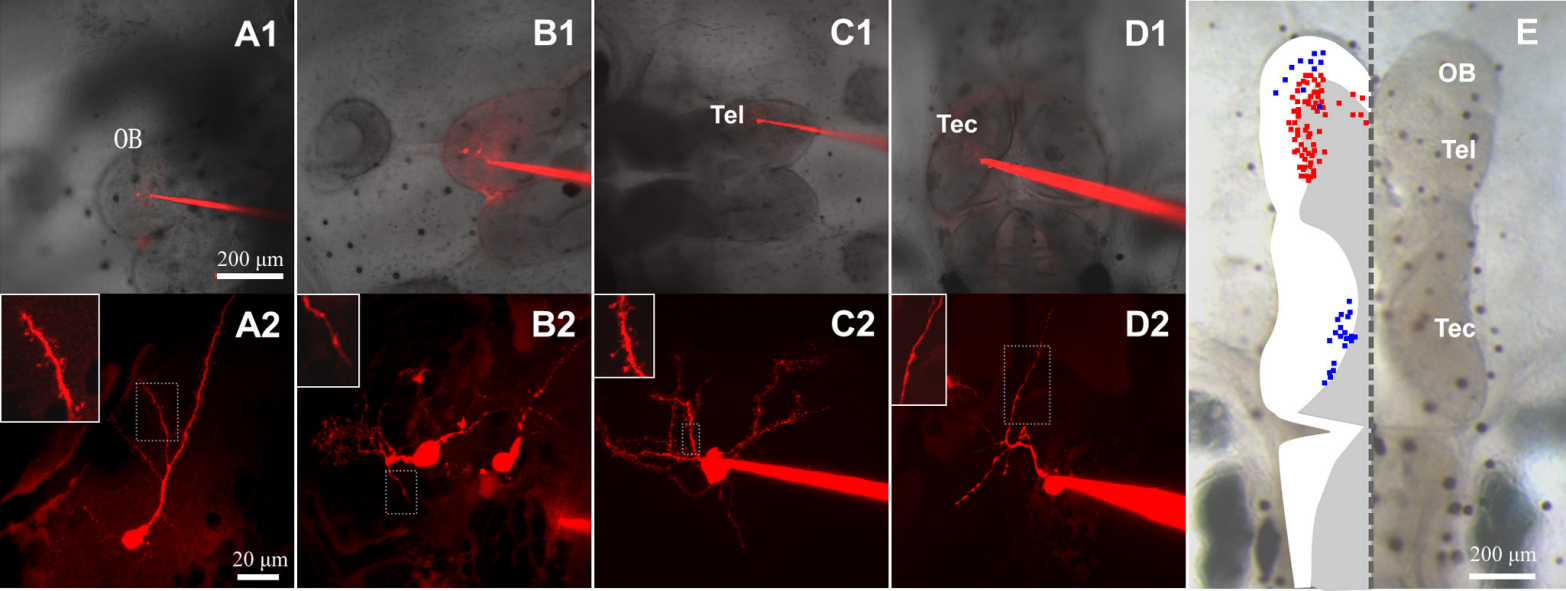
Distribution of spiny neurons in the *Xenopus* tadpole brain. Fluorescent dye was electroporated into neurons located in different brain regions. (A1, A2) Spiny neuron in the OB. (A1) Electrode filled with fluorescent dye inserted into the OB, pointing to a labeled neuron. (A2) High magnification of the neuron in A1. Inset shows spines on a dendrite. (B1, B2) Mitral cell in the anterior OB. (C1, C2) Spiny neuron in the telencephalon (Tel). (D1, D2) Non-spiny neuron in the tectum (Tec). (E) Diagram summarizing all labeled spiny (red) and non-spiny (blue) neurons in different brain areas in the left hemisphere. Twenty neurons randomly labeled in the optic tectum were non-spiny neurons; 69 neurons in the OB and telencephalon were spiny neurons except for 11 neurons.

Currently, the granule cell is the only reported spiny neuron based on morphology in *Xenopus* OB slice [31, 34], and little is known about other types of spiny neuron. To survey the distribution of spiny neurons in the *Xenopus* brain, *in vivo* single-cell electroporation was used to label neurons randomly with fluorescent dye in the OB, telencephalon, and tectum (Fig 1). Neurons with or without spines could be distinguished (Fig 1A2, 1B2, 1C2 and 1D2). All randomly labeled spiny neurons are summarized in a diagram, in which spiny neurons (red) were mainly distributed in the OB and telencephalon, while none was detected in the tectum (Fig 1D). Spiny neurons located in the posterior aspect of the OB were in the granule cell layer, adjacent to mitral cells that are projection neurons without spines distributed in the mitral cell layer. Layering of granule cells and mitral cells in the diagram is coincident with previous reports [45, 46].

### DsRed2 and PSD95-GFP double-labeled spiny neurons in the OB

**Fig 2:**
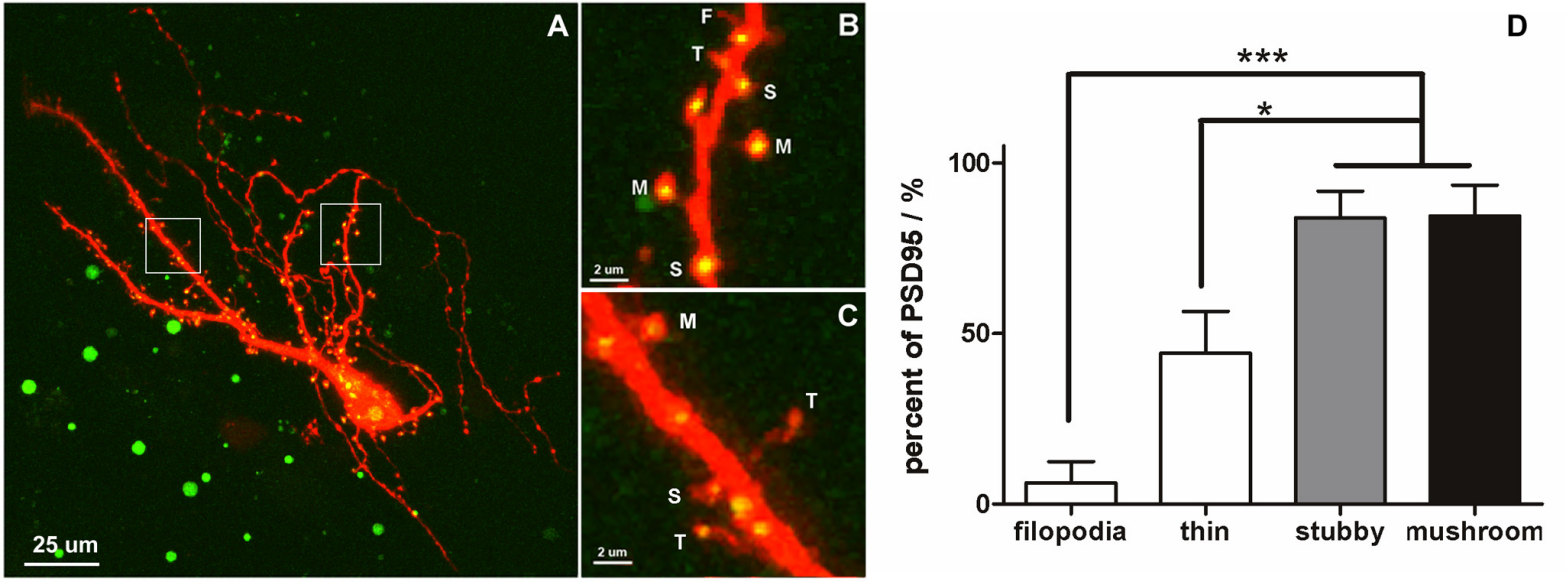
*In vivo* images of DsRed2 and PSD95-GFP double-labeled spiny neurons in *X*. *laevis* tadpoles. (A) A spiny neuron was labeled by DsRed2 and PSD95-GFP. The postsynaptic specializations on spines are shown as yellow puncta. Green dots are developing melanocyte pigmentation not fully inhibited by phenylthiocarbamide (PTU). (B, C) High magnification views showing the different types of dendritic spines of the spiny neuron in (A). F: filopodia; T: thin spine; S: stubby spine; M: mushroom spine. The yellow puncta represent synaptic contacts, most of which were on stubby and mushroom spines. (D) Statistical analysis of the percentage of PSD95-GFP on different types of dendritic spines. Stubby and mushroom spines exhibited a higher number of PSD95-GFP puncta than filopodia and thin spines. Bars indicate mean ± SEM. N = 4 neurons with 9 dendrites were used for colocalization analysis of PSD95-GFP. The significance levels were*P < 0.05, **P < 0.01, and ***P < 0.001.

It has been reported that granule cells in the OB are spiny neuron [31, 34], however, detailed information such as spine morphology, spine type, and dynamic features, is rare known. To examine spine morphology and categories, plasmids PDS95-GFP and DsRed2 were electroporated into olfactory spiny neurons, and *in vivo* time-lapse confocal imaging was performed to visualize dendritic arbors and postsynaptic sites on dendritic spines (Fig 2A). PSD95-GFP is a postsynaptic marker containing a chimeric gene encoding GFP and PSD95 [47, 48] and has been widely used to visualize postsynaptic specializations [41, 49]. DsRed2 was well expressed in spiny neurons of the OB (Fig 2A) and provided clear morphological details of dendritic branches and spines. After overlaying with DsRed2, PSD95-GFP appeared as small yellow puncta distributed over the soma, dendritic trunks, and dendritic spines (Fig 2B and 2C).

Dendritic spines have several different morphological types. The size of the spine head is proportional to the area of postsynaptic density (PSD), the number of postsynaptic receptors, and the number of presynaptic docked vesicles [11]. PSD95-GFP colocalization differed with spine morphology. Confocal images showed that most of the yellow puncta were present on the majority of stubby spines (83.9 ± 7.8%, n = 348) and mushroom spines (84.5 ± 8.9%, n = 388), whereas only a fraction of the small spines contained the PSD95-GFP label (6.3 ± 6.3%, n = 39 for filopodia; 44.2 ± 12%, n = 120 for thin spines; Fig 2D). Statistical analysis showed that the percentage of stubby and mushroom spines with PSD95-GFP puncta was significantly higher than that of filopodia and thin spines (*P* < 0.001; Fig 2D). No significant difference was observed between PSD95-GFP on stubby and mushroom spines. These results indicate that the different morphologies of the spines on OB interneurons of *Xenopus* have different probabilities of forming synaptic connections; small spines are less likely to have synaptic connections, whereas large spines are more likely to have synaptic connections. Thus, like classic spine morphology, PSD95 colocalization confirmed that spiny neurons in the *Xenopus* OB exhibit different spine categories.

To explore the stability and dynamics of each type of dendritic protrusion, images of the olfactory interneurons under short-term observation at 15-min and 2-h intervals were obtained. The stability and dynamics of the 4 different types of spines over these two different time scales were calculated.

### Short-term spine stability and dynamics

**Fig 3:**
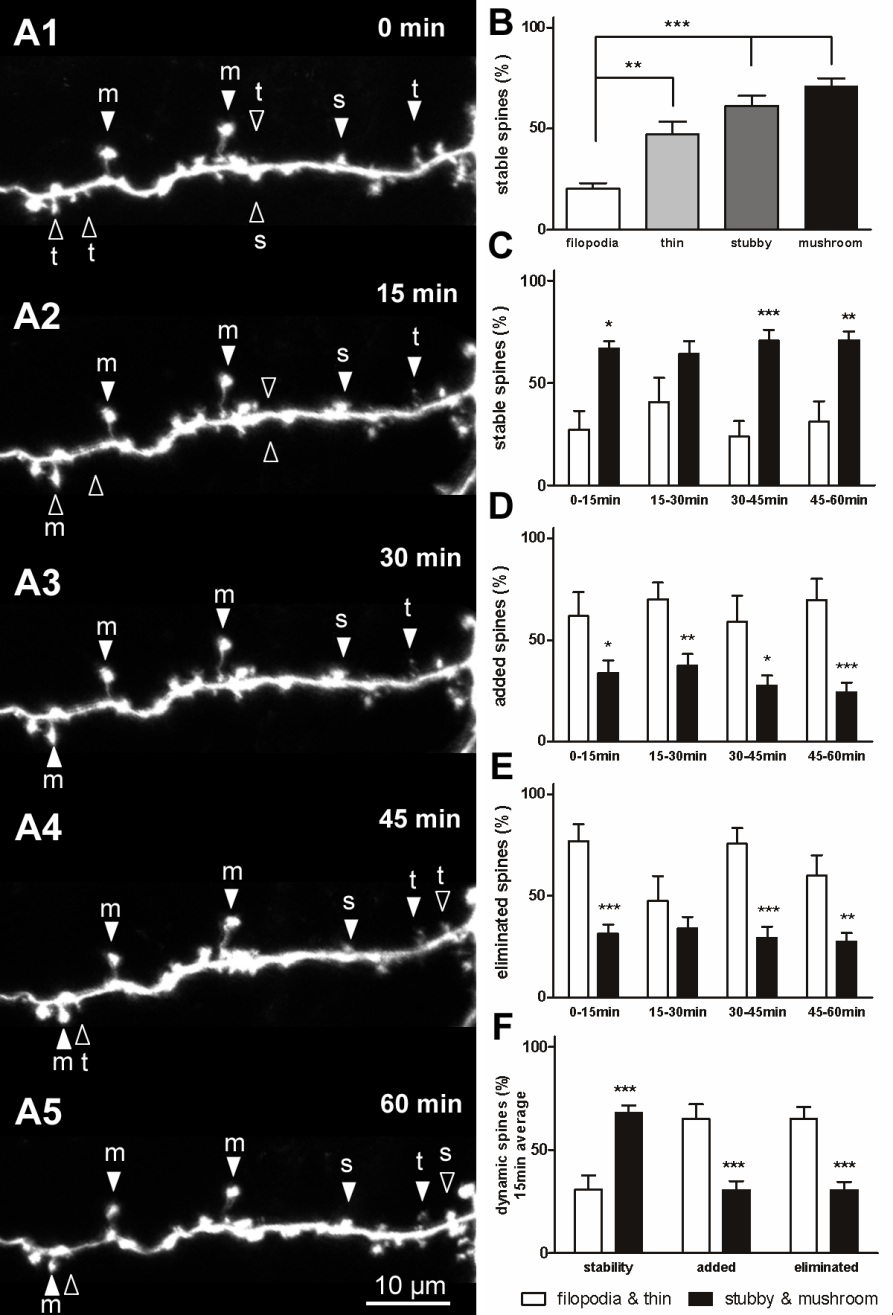
Stability and dynamics of dendritic spines during short-term observation. (A) Serial time-lapse images of a single neuron showing stable spines (filled arrowheads) and dynamic spines (open arrowheads) at 15-min intervals. (B) Stability of the 4 types of dendritic spines was observed at 15-min intervals. The stubby and mushroom spines were more stable than the filopodia and thin spines. (C) Detailed analysis of the stability of filopodia and thin spines versus stubby and mushroom spines at each 15-min interval observation. (D, E) Detailed analysis of filopodia and thin spines versus stubby and mushroom spines that were added (D) or eliminated (E) during each 15-min interval observation. (F) Average dendritic spine stability and dynamics at each 15-min observation. Bars indicate means ± SEM. N = 4 neurons with 9 dendrites were used for the 15-min short-term observation. Significance was set at *P < 0.05, **P < 0.01, and ***P < 0.001.

Many spines remained stable during the short-interval observations (Fig 3A serial). The stability and dynamics of each type of spine were calculated. Filopodia were the most unstable protrusions among the 4 types of dendritic spine, with stability only at 20.2 ± 2.8% (n = 39), which was markedly lower than that of the stubby (61.1 ± 5.1%, n = 328, *P* < 0.001) and mushroom spines (71 ± 3.9%, n = 356, *P* < 0.001; Fig 3B). The stability of the thin spines was higher than filopodia (47.1 ± 6.3%, n = 178, *P* < 0.01), which was also significantly lower than that of the mushroom spines (*P* < 0.05; Fig 3B). A detailed analysis of stability was conducted at each time point. Data show that stability of the large spines was significantly higher than that of the small spines (Fig 3C). The filopodia and thin spines were more dynamic than the stubby and mushroom spines. A higher number of small spines tended to be added or eliminated compared with the large spines (Fig 3D and 3E). A significant difference in addition was observed during the 15–30-min, 30–45-min, and 45–60-min periods (*P* < 0.05; Fig 3D), and a significant difference in elimination was observed during observation intervals (Fig 3E). On average, the percentage of small spine additions or eliminations at each 15-min interval observation was markedly higher than that of the large spines (*P* < 0.001; Fig 3F).

### Long-term spine stability and dynamics

**Fig 4:**
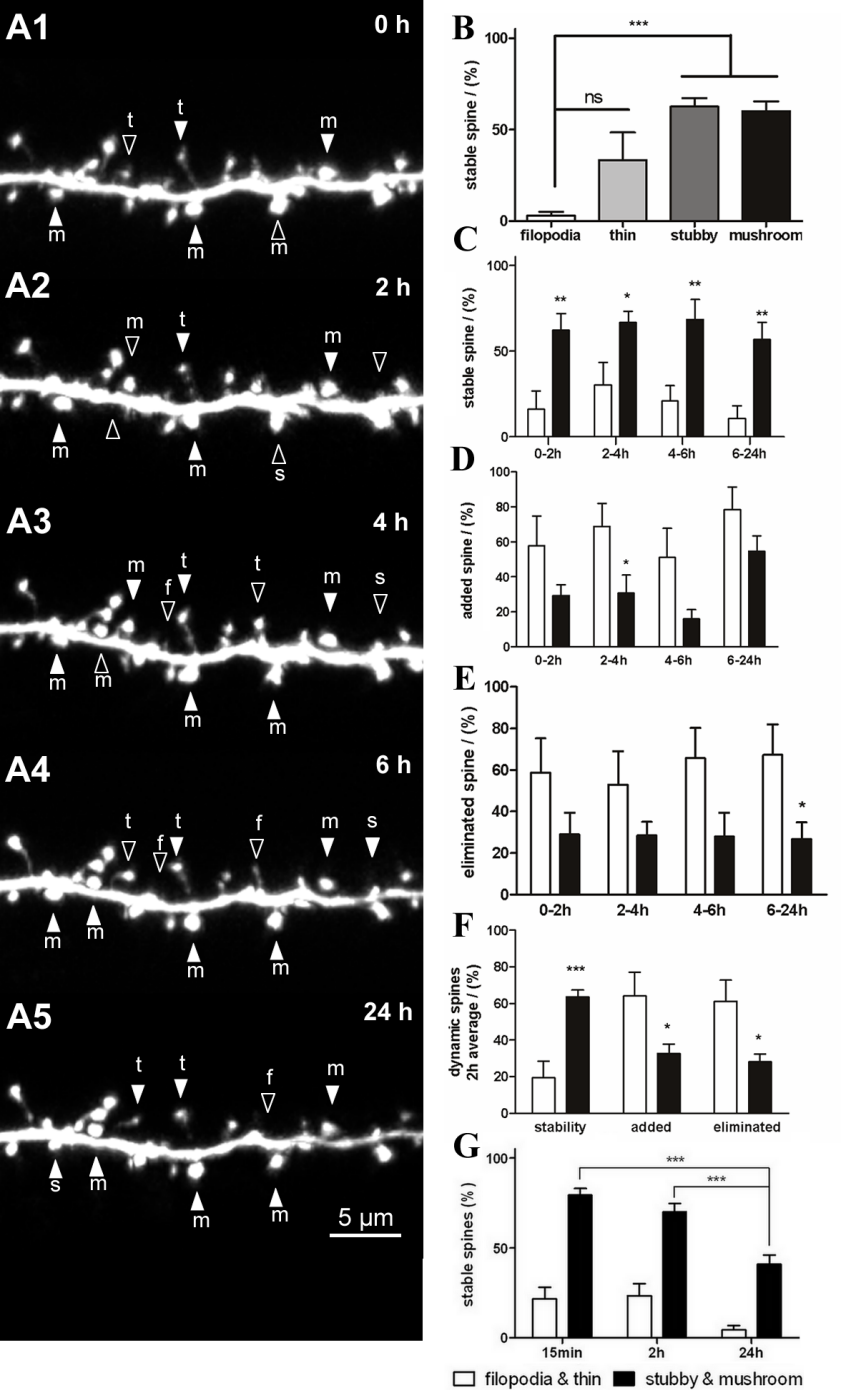
Stability and dynamics of dendritic spines during long-term observation. (A) Serial time-lapse images of a dendritic branch showing stable spines (filled arrowheads) and dynamic spines (open arrowheads) at 2-h intervals. (B) Stability of the 4 types of dendritic spines was observed at 2-h intervals. (C) Detailed analysis of stability of filopodia and thin spines versus stubby and mushroom spines during each 2-h interval observation. (D, E) Detailed analysis of filopodia and thin spines versus stubby and mushroom spines that were added (D) or eliminated (E) during each 2-h interval observation. (F) Average data of dendritic spine stability and dynamics at each 2-h observation. (G) Stability of the dendritic spines at different time interval observations. Bars indicate means ± SEM. N = 4 neurons with 7 dendrites were used for the 2-h and 24-h long-term observations. Significance was set at *P < 0.05, **P < 0.01, and ***P < 0.001.

Long-term stability of up to 24 h at 2-h intervals was observed (Fig 4A serial). The results were similar to those of the 15-min interval imaging. The stability of the filopodia (3.1 ± 2.1%, n = 45) was lower than that of the thin spines (33.4 ± 15.2%, n = 124; Fig 4B) and significantly lower than that of the stubby and mushroom spines (62.7 ± 4.7%, n = 167 and 60 ± 5.4%, n = 176, respectively, *P* < 0.001; Fig 4B). The stability of the thin spines was also lower than that of the mushroom spines (*P* < 0.05; Fig 4B). Detailed analysis at each observation point shows that the stability of the large spines was significantly greater than that of the small spines (0–2 h, 4–6 h, 6–24 h: *P* < 0.01; 2–4 h: *P* < 0.05; Fig 4C). In general, dynamic changes in the filopodia and thin spines were higher than in the stubby and mushroom spines (Fig 4D and 4E). The addition of small spines was higher than that of the large spines during the 0–2-h, 2–4-h, and 6–24-h periods (Fig 4D). The elimination of small spines was also significantly higher than that of large spines during 6–24-h (*P* < 0.05) period (Fig 4E). The average data obtained from 2-h observations showed that small spines were more frequently added or eliminated compared with large spines (*P* < 0.05; Fig 4F). These data indicated that stubby and mushroom spines were more stable and less dynamic than filopodia and thin spines both in the short- and long-term observations. Furthermore, the stability of large spines gradually decreased with longer observation intervals, with significant differences observed at 24-h intervals compared with 15-min and 2-h intervals (*P* < 0.001; Fig 4G).

### Morphological transitions during spine development

**Fig 5:**
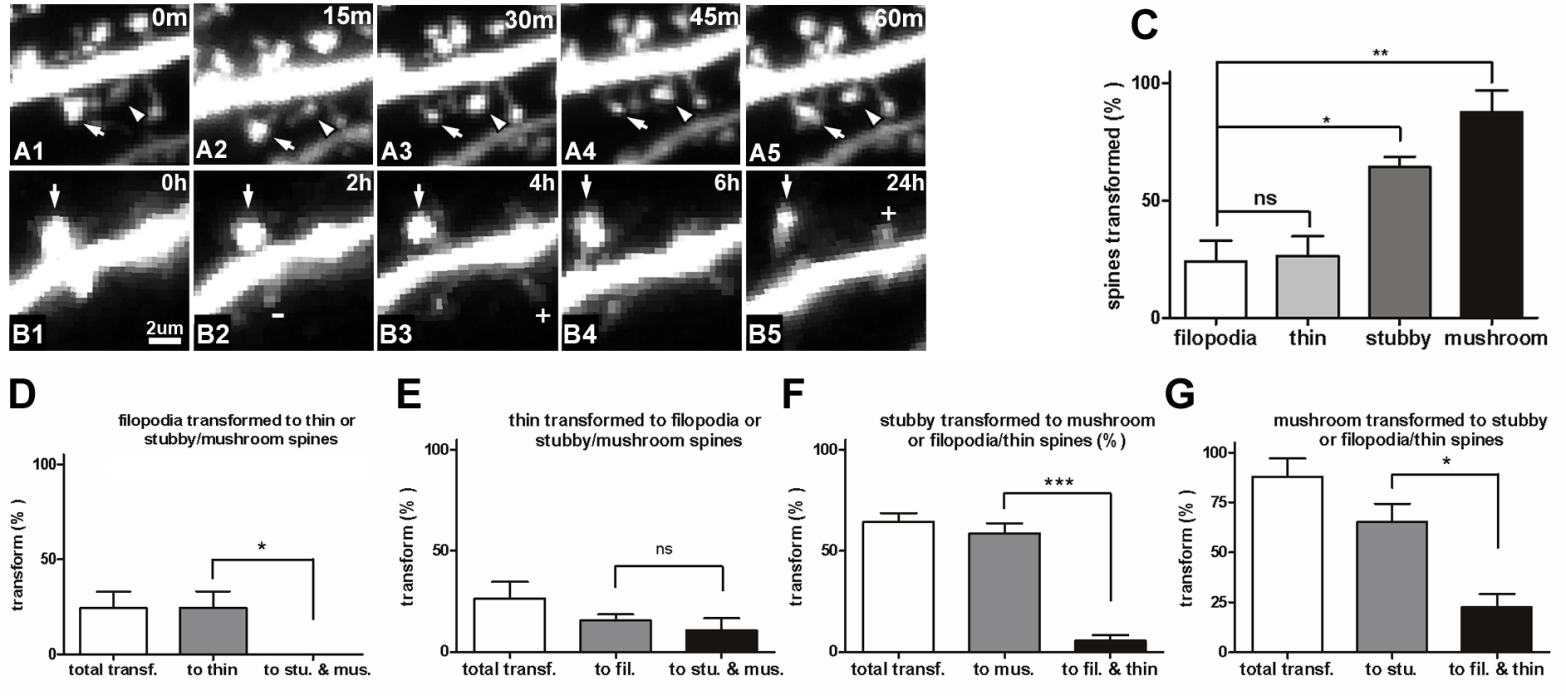
Transformation of dendritic protrusions into other spine types. (A) A mushroom spine (indicated by an arrow in A1) transformed into a thin spine at 30 min (A3), and reverted to a mushroom spine at 60 min (A5). Another thin spine (indicated by an arrowhead) transformed into a mushroom spine at 30 min (A3). (B) A stubby spine (indicated by an arrow in B1) transformed into a mushroom spine at 2 h (B2), remained in that shape up to 6 h (B4), and became a thin spine at 24 h (B5). “+” indicates newly added spines, and “-” indicates eliminated spines. (C) The percentage of spines that transformed into other forms during the pooled data of 15-min and 2-h interval observations. (D–G) Percentage of each spine type that transformed into other types. (D) All filopodia transformed into thin spines, and a few transformed into large spines (*P* < 0.05). (E) Some changed thin spines transformed into filopodia, and some transformed into large spines; no significant difference was observed between these two forms. (F) Most of the stubby spines transformed into mushroom spines, and only a few transformed into small spines (*P* < 0.001). (G) Most of the mushroom spines transformed into stubby spines, and only a few transformed into small spines (*P* < 0.05). Figure labels: trans. = transformation, fil. = filopodia, stu. = stubby, and mus. = mushroom. Neurons used for transformation analysis were from the 15-min short-term and 2-h middle-term observations. Bars indicate means ± SEM. Significance was set at *P < 0.05, **P < 0.01, and ***P < 0.001.

During development of dendritic spines, most spines exhibit structural plasticity with rapid extension or retraction [1, 7]. Thus, what are the preferences when spines transform morphology from one to other categories? Fig 5A (serial) shows a mushroom spine indicated by an arrow at 0 min (Fig 5A1), that transformed into a thin spine at 30 min (Fig 5A3) and back to a mushroom spine at 60 min (Fig 5A5). Another thin spine indicated by an arrowhead at 0 min (Fig 5A1) transformed into a mushroom spine at 30 min (Fig 5A3). Fig 5B (serial) shows a stubby spine indicated by an arrow (Fig 5B1) that transformed into a mushroom spine at 2 h (Fig 5B2) and remained so at 6 h (Fig 5B4) before becoming a thin spine at 24 h (Fig 5B5). The frequency with which spines disappeared or transformed into other types was subsequently determined. We found that small spines were more likely to disappear entirely, whereas large spines were more likely to change shape rather than disappear. Pooled data from the 15 min and 2 h time courses show that only 24.2 ± 8.9% (n = 56) of filopodia transformed into thin spines, and 26.4 ± 8.3% (n = 67) of thin spines changed their morphology (Fig 5C). These values were both significantly lower than the percentages for the stubby (64.3 ± 4.5%, n = 376) and mushroom (87.9 ± 9.1%, n = 333) spines (P < 0.01; Fig 5C). The percentage of specific spine types that transformed into other types was then obtained. Results showed that all changed filopodia transformed into thin spines compared with large spines (P < 0.05; Fig 5D). However, no significant difference between thin spines that transformed into filopodia and those that became large spines (15.7% and 10.8%, respectively) was observed (Fig 5E). A few large spines transformed into small spines (Fig 5F and 5G). Most of the changed stubby spines transformed into mushroom spines (58.7%) rather than small spines (5.6%, P < 0.001; Fig 5F). Similarly, most of the changed mushroom spines transformed into stubby spines (66.3%) rather than into small spines (26.6%, P < 0.05; Fig 5G). These results indicate that spines have diverse preferences during morphological transitions. Large spines are more likely to preserve their synaptic connections within stubby and mushroom spines rather than disappearing entirely as small spines. In the small spine category, almost all unstable filopodia transform into thin spines, whereas only a small number of changed thin spines develop into large spines, indicating that some of the large spines originate from small spines during development.

### Spine stability after olfactory nerve severance

**Fig 6:**
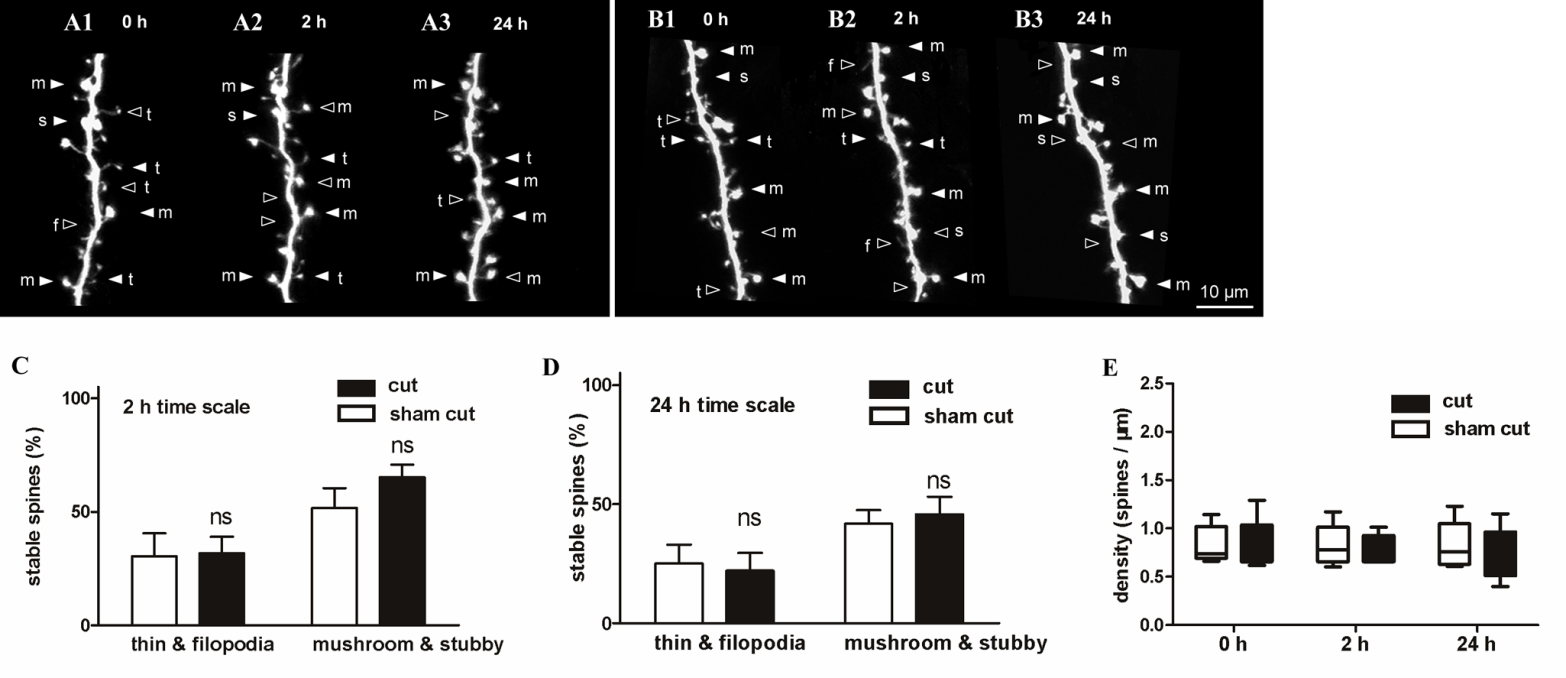
Stability of dendritic spines after olfactory nerve severance. (A, B) Serial time-lapse images showing the morphology of dendritic spines in the sham group (A) and olfactory nerve severance group (B) imaged at 0 h, 4 h, and 24 h, respectively, after surgery. (C, D) Stability of small and large spines after sensory deprivation compared with normal spines during the 2-h and 24-h observation periods. (E) Density of the spines after surgery. Bars indicate means ± SEM. N = 10 neurons with 16 dendrites were used for the sensory deprivation analysis. Significance was set at *P < 0.05, **P < 0.01, and ***P < 0.001.

Maturation of the nervous system is experience-dependent, and the patterns of neuronal connectivity are affected by activity [3]. Experimental evidence indicates that experience and neuronal activity are key to spine dynamics and cortical plasticity [1, 2, 9, 50, 51]. To observe spine dynamics of OB interneurons after activity blockade, olfactory nerves were severed to prevent olfactory sensory input. To confirm severing of the olfactory nerves, 1% DiI was injected into the OB for retrograde tracing of the olfactory nerves. After severance, disconnection of the DiI-labeled olfactory nerve was clearly demonstrated (S1 Fig). Stage 46/47 tadpoles with single labeled olfactory interneurons were randomly divided into sham cut and cut groups. In each group, the surgical procedure was the same except that olfactory nerves were not severed in the sham cut group. After nerve severance, tadpoles were imaged at 0 h (Fig 6A1 and 6B1), 2 h (Fig 6A2 and 6B2), and 24 h (Fig 6A3 and 6B3). Stability of the small and large spines observed at the 2-h interval was similar to the spines in the control group (Fig 6C). The 24 h interval observation also showed no significant difference between the two groups (Fig 6D). In addition, data showed that spine density did not change within 2 h and 24 h (Fig 6E) after olfactory nerve severance compared with the control group.

## Discussion

### The *X*. *laevis* tadpole as an *in vivo* model for studying spine dynamics

Spiny neurons are rarely found in non-vertebrates such as *Drosophila melanogaster*, *Caenorhabditis elegans* [52], and even in non-mammalian vertebrate models such as zebrafish and *Xenopus*. According to studies on olfactory bulb slices, granule cells are the only spiny neurons in *Xenopus* [31, 34], while in zebrafish, a lower vertebrate, spiny neurons have been scarcely reported except for spine-like or knob-like protrusions on Mauthner axons [16, 27]. This implies that spines may have evolved as complex structures to execute higher-level functions in more advanced nervous systems.

In the present study, a distribution map of spiny neurons was drawn based on random fluorescent dye labeling in the OB, telencephalon, and tectum. Spiny neurons were widely found in the OB and extensively distributed in the telencephalon (Fig 1), but were not detected in the optic tectum, which is consistent with previous studies of tectal neurons [41]. However due to technical limitation of single-cell electroporation and imaging depth, it was difficult to label neurons in all brain areas. Thus, only a subset of neurons was surveyed.

Granule and mitral cells are the primary neurons in the *Xenopus* OB. Mitral and periglomerular cells are located in the anterior and granule cells in the posterior area of the OB. Our characterization of spiny and non-spiny neurons in the OB is consistent with previous studies [34, 45, 46, 53]. Spiny neurons labeled for *in vivo* imaging were granule cells as they were found mainly in the granule cell layer (Fig 1D), and they were rarely labeled in the mitral cell layer. Furthermore, mitral and granule cells differ in morphology. Mitral cells are projection neurons without spines, and their terminals form tufts, a characteristic branching structure, while granule cell dendrites have a typical appearance of spines [34]. In rodents, granule cells are located in the deepest layer of the OB (~350 μm below the dorsal surface of the OB in mouse). This makes it difficult to label and image them and only apical dendrites have been visualized with limited optical resolution [54]. Thus, *Xenopus* tadpole provides unique advantages for studying granule cells in the OB. In addition to the OB, spiny neurons were extensively distributed in the telencephalon, possibly providing another *in vivo* model for studying spines; however, studies on the telencephalon are currently limited in *Xenopus*.

In the OB of the *Xenopus* tadpole, all layers of the developing OB are present at stage 44. The mature laminar structure of the OB appears at stage 48/49 and remains constant throughout larval life even into adulthood, and increases in size [55]. Our preliminary experiments showed that interneurons have a few spines at stage 44/45, becoming more abundant at stage 46/47. Thus, tadpoles at stage 46/47 were selected for spine dynamics and stability analysis. After stage 50/51, tadpoles gradually lose their optical transparency. Hence, we could not visualize spines in the more mature brain.

### Spine categories and structural plasticity

PSD95 is a postsynaptic scaffold protein that plays a substantial role in synapse maturation; it clusters NMDA receptors on the postsynaptic membrane [56, 57]. PSD95-GFP has been widely used as a postsynaptic marker to visualize postsynaptic specializations [47, 48, 58, 59]. In a previous study, PSD95-GFP was expressed in *Xenopus* tectal neurons and was localized to the postsynaptic side of the mature synapse with clearly defined postsynaptic specializations [41]. These studies confirmed that PSD95-GFP is recruited to synapses and validated its use as a marker to visualize postsynaptic sites *in vivo*. Our data show that distribution of PSD95-GFP varied across different types of spines. Large stubby and mushroom spines showed higher PSD95-GFP colocalization compared with small filopodia and thin spines. This result is consistent with previous findings that most filopodia do not form mature synaptic contacts with presynaptic axons, and that a higher number of synaptic vesicles are present in the presynaptic membrane of stubby and mushroom spines [8, 60, 61]. Thus, the spiny neurons that we observed in *Xenopus* OB fell into classic categories according to morphology and correlation with PSD95. Dendritic spine stability was spine type-dependent in the *Xenopus* tadpole. Filopodia and thin spines showed low stabilities (Figs 3B and 4B). Synaptic connections that form on filopodia lack AMPA receptors and are always in a state of continuous formation and elimination [12, 13, 62]. Stubby and mushroom spines exhibited higher stabilities at ~70–80%. Recently, adult-born interneurons in mouse OB were successfully labeled by viral infection and observed through two-photon imaging [54]. However, granule cells in the developing OB were rarely observed due to their deep location. Our data revealed that synaptic connections of OB granule cells were highly dynamic, with continuous formation and elimination. Small filopodia and thin spines represented immature and exploratory synaptic connections. Large spines represented more stable about 75% were morphologically consistent (Figs 3D–3F and 4D–4F). In the mouse OB, 12-h and 24-h interval observations showed that about 65% and 55% of interneuron spines, respectively, were stable, and that they were the most plastic neurons [54], while other cortical spines were stable for weeks to months [10, 12, 14]. These data indicate that during development, synaptic connections on granule cells are dynamic, similar to adult-born interneurons in the mouse OB [54].

### Morphological transitions during spine development

*In vivo* observations have shown that dendritic spines were highly motile [1, 14, 63], and spines were classified into only two types, spine and filopodia, rather than 4 types (mushroom, stubby, thin, and filopodia). Therefore, details of morphological transitions are limited in previous reports. We observed morphological transitions among all spine categories and found that different spines have diverse preferences during morphological transitions from one spine type to others. Large spines are more likely to preserve synaptic connections between mushroom and stubby spines. Most small spines disappear directly or transform between filopodia and thin spines, and only a small number of thin spines develop into large spines. The morphological transitions are thought to be directly controlled by actin, which is a highly dynamic and concentrated component in the spine and regulates spine protrusion and retraction bidirectionally. Spine dynamic changes are tightly correlated with synaptic architectures [64, 65]. In addition to subcellular factors of neurons, spine motility is also developmentally regulated, as fewer transitions occur in mature neurons than in developing neurons [11].

### Dendritic spine stability after olfactory nerve severance

After blocking the input from mitral/tufted cells, dendritic spine stability was not affected and the 4 spine types showed little change, which suggests that sensory input has limited effects on interneuron dendritic spines in the developing tadpole. Similar data were obtained from adult-born interneurons in the mouse OB after odor deprivation [54]. Another *in vivo* study indicated that in the barrel cortex, spine motility was sensitive to input deprivation during a critical period, but had no effect in younger or older animals [1]. Although we observed that, olfactory nerve severance had limited effects on interneuron dendritic spines, it is possible that spines are regulated by development rather than sensory experiences as shown in previous reports [7, 11]. Our data support a model in which dendritic dynamics of *Xenopus* OB neurons are comparable to those of mammalian cortical neurons.

In the present study, instead of using a pharmacological method to block activity, an olfactory nerve severance was performed to block input from mitral/tufted cells. For the aquatic *Xenopus* tadpoles with extremely small brains, it is difficult to achieve specific blocking effects using pharmacological manipulations such as TTX delivery due to diffusion to other tissues.

This study in *Xenopus* established a classic but new animal model for spine studies. It could be widely applied in spine research, such as spinogenesis, spine pruning, spine plasticity, and spine pathology in brain disorders.

## Supporting Information

S1 Fig Olfactory nerve severance. A small amount of 1% DiI was injected into the olfactory bulb for retrograde tracing of the olfactory nerves. Severance was performed under bright field and confirmed under a fluorescence microscope (A, C). Intact olfactory nerves and completely disconnected olfactory nerves (C, D).
(TIF)
