# Supplementary figures and images for: In Vivo Study of Dynamics and Stability of Dendritic Spines on Olfactory Bulb Interneurons in Xenopus laevis Tadpoles
Source: PLoS One. 2015 Oct 20;10(10):e0140752. doi: 10.1371/journal.pone.0140752 (PMC4617280; doi:10.1371/journal.pone.0140752)

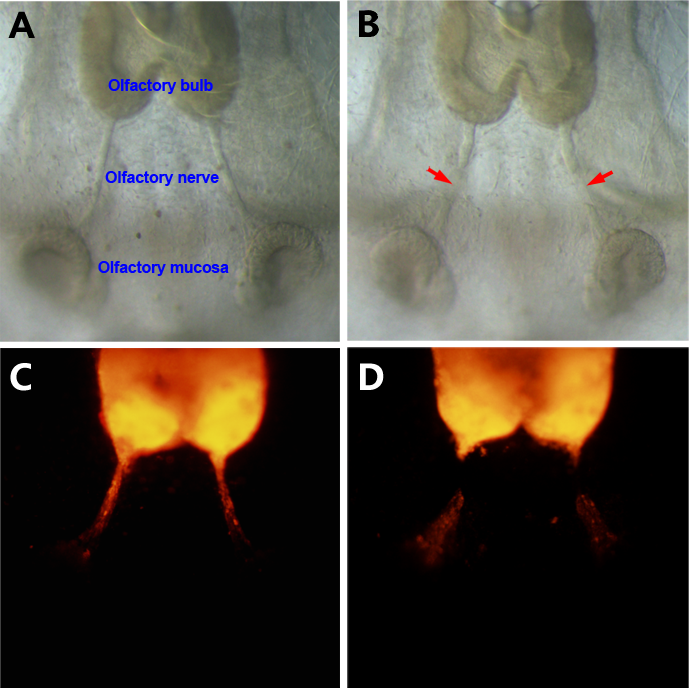

Supplement: S1 Fig — A small amount of 1% DiI was injected into the olfactory bulb for retrograde tracing of the olfactory nerves. Severance was performed under bright field and confirmed under a fluorescence microscope (A, C). Intact olfactory nerves and completely disconnected olfactory nerves (C, D). (TIF) [file pone.0140752.s001.tif]
